# Pyelonephritis Caused Solely by *Escherichia hermanii*

**DOI:** 10.5812/jjm.18138

**Published:** 2014-05-01

**Authors:** Yan Qing Tong, Bing Xin, Shu Qing Sun

**Affiliations:** 1 Department of Nephrology, The First Affiliated Hospital to Changchun University of Chinese Medicine, Changchun City, China; 2 Department of Microbiology, The First Affiliated Hospital to Changchun University of Chinese Medicine, Changchun City, China

**Keywords:** *Escherichia*, Urinary Tract, Gram-Negative Bacterial Infections

## Abstract

**Introduction:**

In contrast with *Escherichia coli*, the association of *E. hermanii* with urinary tract infections has not been described.

**Case Presentation:**

In this case, *E. hermanii* was the sole isolate recovered from urine specimens of a pyelonephritis patient. The organism was found to be susceptible to piperacillin-tazobactam, ceftazidime, cefazolin, cefixime, aztreonam, gentamicin, tobramycin, imipenem, meropenem and amikacin, and resistant to amoxicillin. Antibiotic treatment was initiated with oral cefixime (400 mg every 24 hours). The symptoms were relieved within 72 hours after therapy. A urine sample was taken seven days after antibiotic therapy. *E. hermanii* was no longer isolated.

**Discussion:**

The present case demonstrates that the uropathogenic *E. hermanii* clone can cause destruction of the kidneys. During asymptomatic bacteriuria or cystitis, the bacteria remain in the urinary tract. Even when pyelonephritis develops, inflammatory response of the host is still restricted to the urinary tract. These signs mean that uropathogenic *E. hermanii* may be not very virulent.

## 1. Introduction

Pyelonephritis represents a renal injury induced by recurrent or persistent renal infection, associated with progressive renal scarring and may lead to end-stage renal disease (1). In 1982, *Escherichia hermanii* was classified as a distinct species within the *Escherichia* genus on the basis of DNA–DNA relatedness (2) and was considered to be nonpathogenic. In contrast to *E. coli*, the association of this organism with urinary tract infections has rarely been described. In this report, we described some of the uropathogenic properties of *E. hermanii*, which was clinically isolated from the urine of a pyelonephritis patient.

## 2. Case Presentation

A 65-year-old Asian female (Height: 5 feet 4 inches; Weight: 52.5 kg; Occupation: retired teacher) referred to our clinic March 8th, 2013. Her family and social history was as follows; she lived alone and had quit cigarette smoking 10 years ago, with no alcohol or illicit drug use. Her mother had a history of colon cancer. She had a past medical history of a left breast mass that had required resection and radiation therapy. Three weeks before being admitted, the patient had presented an initial episode of cystitis. This was treated by a single dose of fosfomycin, which led to regression of the clinical symptoms. One day before hospitalization, she presented sudden onset of clinical signs of pyelonephritis: dysuria, urgency, frequency, urinary incontinence, suprapubic pain and elevated temperature (37.7°C). Urinalysis revealed positive leukocyte esterase, with 50-60 white blood count and 18-22 red blood count per high-power field. Her serum creatinine level was 125.3 umol/L and hemoglobin level was 10.7 gr/dL. There were no other significant laboratory findings.

Urine samples were taken and cultured using both Columbia blood agar (Acumedia Manufacturers, Inc., US.) and MacConkey agar (Teknova Inc., US.). Microscopic examination of Gram stained smears of the urine revealed Gram-negative rods. Yellow colonies of the microorganism were detected on all culture media after overnight incubation. No other bacteria were detected. The isolate was identified as *E. hermanii* after assessment using the API 20E system (bio-Merieux, Lyon, France) for biochemical properties and enzyme activities. 

This isolate was forwarded to the local laboratory and biochemical testing and sequencing of the 16S rRNA gene confirmed the isolate as *E. hermanii*. The upstream primer used for the amplification of the 16S region in the *E. hermanii* genome was 5′-AGAGTTTGATCCTGGCTCAG-3′. The downstream primer was 5′-ACGGCTACCTTGTTACGACTT-3′. The nucleotide sequence of the 16S rRNA gene was 99.86% identical to that of *E. hermanii* T91, according to the NCBI GenBank database. Bacterial cultures were grown aerobically at 37°C in an incubation chamber.

Antimicrobial susceptibility testing was performed using minimum inhibitory concentration (CLSI) broth microdilution minimum inhibitory concentration (MIC) method (3). The organism was found to be susceptible to piperacillin-tazobactam (MIC ≤ 4 mg/L), ceftazidime (MIC ≤ 1 mg/L), cefazolin (MIC ≤ 4 mg/L), cefixime (MIC ≤ 0.25 mg/L), aztreonam (MIC, 4 mg/L), gentamicin (MIC ≤ 4 mg/L), tobramycin (MIC ≤ 1 mg/L), imipenem (MIC, 2 mg/L), meropenem (MIC ≤ 0.25 mg/L) andamikacin (MIC ≤ 1 mg/L); and resistant to amoxicillin (MIC ≥ 16 mg/L). Antibiotic treatment was initiated with oral cefixime (400 mg every 24 hours). The symptoms were relieved within 72 hours after therapy. A urine sample was taken seven days after antibiotic therapy. E. hermanii was no longer isolated.

## 3. Discussion

Pyelonephritis is a particular type of Urinary tract infection (UTI) that commonly originates in the urethra or bladder and travels up into kidneys. Pyelonephritis needs rapid medical care. If not treated accurately, the infection can permanently damage the kidneys or the bacteria can spread to the bloodstream and cause a life-threatening infection. Pyelonephritis is a severe form of UTI with women being more likely to be affected than men. A wide variety of bacterial genera including enteric bacteria have been identified as putative pathogens. *E. coli *is involved in most UTI cases: >80% of community-acquired UTIs and approximately 50% of UTIs in hospital patients (4). 

Although other bacteria belong to the family *Enterobacteriaceae*, such as *Proteus* spp., and *Klebsiella pneumoniae* are occasionally isolated from the urinary tract, the association of *E. hermanii* with pyelonephritis has rarely been reported. Artero et al. (5) recently described *E. hermanii* isolated from the urine of a pregnant woman with pyelonephritis from the United States in a retrospective analysis of archived isolates. However, the origin of these strains was unclear and no clinical information was provided in the report.

*E. hermanii* is an extremely rare etiological agent for invasive infections. As a member of the family *Enterobacteriaceae *(6), *E. hermanii* is a Gram-negative rod-shaped bacterium commonly found in the wounds and feces of warm-blooded animals (2). In contrast to *E. coli*, *E. hermanii *can produce a yellow pigment and show various biochemical characteristics including the fermentation of cellobiose and a positive reaction to KCN (2). Unlike *E. coli*, this organism has not been detected in human genitourinary tract thus far. In this report, the organism was isolated from a urine specimen, which provides evidence that *E. hermanii* plays a role as an invasive pathogen in the urinary tract. 

*E. hermanii* is primarily an opportunistic pathogen which causes disease in immunocompromised hosts (e.g. diabetes mellitus, malignancies, extremes of age) or in those who use a central catheter. It has been considered as an associated pathogen in a few invasive infections, which were mostly attributed to other coexisting bacteria that were more pathogenic (7, 8). In this case, *E. hermanii* was the sole pathogen recovered from a patient with pyelonephritis and was isolated in large numbers. However, its pathogenic role in this patient was uncertain.

It is very difficult to definitively comment on the treatment of *E. hermanii* UTIs. Findings and outcomes of the published data about the antimicrobial susceptibilities of clinical *E. hermanii *isolates are limited. However, in this case, *E. hermanii* isolates were susceptible to antimicrobial treatment. The *in vitro* susceptibilities of these isolates showed that piperacillin-tazobactam, ceftazidime, cefazolin, cefixime, aztreonam, gentamicin, tobramycin imipenem, meropenem, and amikacin were active *in vitro*. Isolates also showed a low-level resistance against amoxicillin, similar to that reported by Fitoussi et al. and Beauchef-Havard et al. (9, 10). However, more clinical experiments are mandatory. The mild clinical infection of our patient, allowed an oral regimen. We selected cefixime for its lowest MIC *in vitro* effectiveness against the pathogen.

The present case demonstrates that the uropathogenic *E. hermanii* clone can cause destruction of the kidneys. During asymptomatic bacteriuria or cystitis, the bacteria remain in the urinary tract. If insufficiently treated, the bacteria in the bladder will travel up into the kidneys. Even when pyelonephritis develops, the inflammatory response of the host is still restricted to the urinary tract. These signs mean that uropathogenic *E. hermanii* may not be very virulent.

In conclusion, the case of our study highlights the fact that *E. hermanii* may cause infection in the urinary tract as a sole pathogen. The case report we presented provides further evidence for the uropathogenic potential of *E. hermanii*, although much is still unclear about its pathogenicity.
